# A Trust Evaluation Algorithm for Wireless Sensor Networks Based on Node Behaviors and D-S Evidence Theory

**DOI:** 10.3390/s110201345

**Published:** 2011-01-25

**Authors:** Renjian Feng, Xiaofeng Xu, Xiang Zhou, Jiangwen Wan

**Affiliations:** School of Instrument Science and Opto-electronics Engineering, Beijing University of Aeronautics and Astronautics (Beihang University), Beijing 100191, China; E-Mails: alickelly@gmail.com (X.X.); zhouxiangbuaa@163.com (X.Z.); sensory@buaa.edu.cn (J.W.)

**Keywords:** wireless sensor networks, network security, trust evaluation, node behaviors, evidence theory

## Abstract

For wireless sensor networks (WSNs), many factors, such as mutual interference of wireless links, battlefield applications and nodes exposed to the environment without good physical protection, result in the sensor nodes being more vulnerable to be attacked and compromised. In order to address this network security problem, a novel trust evaluation algorithm defined as NBBTE (Node Behavioral Strategies Banding Belief Theory of the Trust Evaluation Algorithm) is proposed, which integrates the approach of nodes behavioral strategies and modified evidence theory. According to the behaviors of sensor nodes, a variety of trust factors and coefficients related to the network application are established to obtain direct and indirect trust values through calculating weighted average of trust factors. Meanwhile, the fuzzy set method is applied to form the basic input vector of evidence. On this basis, the evidence difference is calculated between the indirect and direct trust values, which link the revised D-S evidence combination rule to finally synthesize integrated trust value of nodes. The simulation results show that NBBTE can effectively identify malicious nodes and reflects the characteristic of trust value that ‘hard to acquire and easy to lose’. Furthermore, it is obvious that the proposed scheme has an outstanding advantage in terms of illustrating the real contribution of different nodes to trust evaluation.

## 1. Introduction

Recent technology development in the fields of wireless communication and MEMS has facilitated the extensive distribution of wireless sensor networks (WSNs) which are reliable, accurate, flexible, inexpensive and easy to deploy. In many applications, for instance, environment monitoring and battlefield spying, the nodes are vulnerable to be attacked by passive eavesdropping, active intrusion, message flooding, fake information inserting, *etc*. Among the above hostile attacks, passive eavesdropping helps adversaries intercept private information. Active intrusion makes it possible for adversaries to delete information, insert false information or impersonate nodes, which destroy the usability, integrality, security certificate and non-reputation of WSNs. Unfortunately, the available complicated encryption algorithms are unsuitable for WSNs because of the restricted capabilities of low cost nodes. Hence, the WSNs usually adopt security method based on symmetric cryptographic methods.

However, the intrusion detection and prevention schemes with cryptographic protection are unable to identify the destructive threat by the authenticated nodes which have been compromised. If the compromised nodes cannot be identified in time, secret information may be revealed and the whole network could be under the control of the adversaries. Therefore, an efficient mechanism is urgently needed to identify the compromised nodes and take measures to minimize the destruction or loss in the network.

Trust management is fundamental to identify malicious, selfish and compromised nodes which have been authenticated. It has been widely studied in many network environments such as peer-to-peer networks, grid and pervasive computing and so on. However, in reality, sensor nodes have limited resources and other special characters, which make trust management for WSNs more significant and challenging. Up to the present, research on the trust management mechanisms of WSNs have mainly focused on nodes’ trust evaluation to enhance the security and robustness. The practical applications of this method include the route, data integration and cluster head vote [1].

Although some existing approaches play good roles in improving security of other networks, trust management in WSNs still remains a challenging field. In this paper, we propose NBBTE algorithm by analyzing the advantages and disadvantages of existed methods [2–24]. Firstly, each node establishes the direct and indirect trust values of neighbor nodes by comprehensively considering various trust factors, and combining these factors together with network security grade, correlation of context time and rewards degree. The trust factors mentioned above include packet receive, send, strictness, delivery, consistency and availability, *etc*. Secondly, fuzzy set theory is used to decide the trustworthiness levels in accordance with the fuzzy subset grade of membership functions. Based on the levels of trustworthiness, the basic confidence function of D-S evidence theory is accordingly formed. Finally, using the revised Dempster rules of combination, the integrated trust value of a node is obtained by integrating its trustworthiness of multiple neighbor nodes.

The remainder of this paper is organized as follows. Section 2 introduces the related work. Section 3 describes the related notions and parameters. In Section 4, the proposed scheme is specifically depicted, including its design idea and practical implement approach. The performance of the scheme is mainly evaluated in Section 5. Finally, in Section 6, we make some concluding remarks.

## 2. Related Work

The trust management methods can be classified into two categories: distributive authorization system based on trust chain and network trust evaluation system based on nodes’ behaviors [2–5]. (1) In the former system, the authorized individual is allowed to collect all the information of other authorized ones. It checks the consistency through strategy inference engine in light of local policy and authorization requirements. In addition, if a trust chain exists between two strange individuals, the authorization is able to be relayed by signing indirect objects which have trust rights. That is to say, the authorization individual has rights to deal with its trusted objects. But it is very dangerous for the limited resources of WSNs when the authorization nodes are compromised. (2) In the latter system, individuals acquire all kinds of related information, including the actions of evaluated individuals, interacting rules and other individuals’ opinions. Then, the sensor nodes obtain other nodes’ trust value by different computing method in application. This trust management method has advantages of less resources consumption, peer-to-peer structure and no centers. Therefore, trust management schemes similar to the latter one are more frequently applied in the WSNs.

Viljanen [6] came up with all kinds of ingredients of trust evaluation after nearly ten years of research on the trust of the network, which has a guiding effect on trust measurement of the sensor nodes in WSNs. Crosby *et al*. [7] propose a trust evaluation model based on the classical probability model, which uses simple statistical methods to accomplish trust value computation without considering the trust recommendation between sensor nodes. Therefore, it cannot reflect nodes’ real-time trust state accurately. Ganeriwal *et al*. [8] make a trust evaluation model and uncertainty analysis based on Bayes theory. Because the lack of prior knowledge about wireless sensor networks, the model’s subjective assumptions of prior distribution aggravates the uncertainty of trust. These two models both regard the subject fuzziness of trust as the randomness and use pure probability statistic method to assess trustworthiness, which is difficult to obtain prior knowledge from practical application and inevitably result in something unreasonable.

In order to deal with the subjective fuzziness of trust evaluation, Tang *et al*. [9] propose a trust evaluation model based on fuzzy logic, which provides a formalized inference mechanism and does not give specific trust calculation methods. Krasniewski *et al*. [10] use the base station to make a centralized trust management of cluster head election. If the cluster head is unbelievable, a new one will be elected in another round to avoid effectively malicious or selfish node to act as cluster head. But the centralized trust management model increases the network communication payload and the passive trust decision-making slows down the convergent speed of cluster head election. Song *et al*. [11] add trust component to the LEACH algorithm, where nodes select the highest trust value one from their neighbors as cluster head. Although the distributed algorithm in this scheme has high convergent speed, reputation-based trust management may be vulnerable to collusion attacking. TRANS [12,13], which is proposed by Tanachaiwiwat *et al*., searches and marks the suspicious positions in WSNs based on the geographic information route. It puts the nodes at the suspicious positions on a black list and broadcasts them to all the other nodes, thereby achieving trust-based secure routing. But there is the possibility that some nodes are misjudged to be malicious because of the abominable channel or compromised nodes. Consequently, it requires a mechanism to allow the nodes in black list to turn into usable nodes again, whereas the model neglects this point. Hur *et al*. [14,15] divide the network into several grids, which accomplishes secure data integration by crosschecking the consistency of nodes’ data, but collusion attacks are not able to be resisted very well.

PTM [16–18], a research sub-item of UBISEC (secure pervasive computing) supported by Europe IST FP6, which builds models mainly in accordance with revised D-S evidence theory, defines the inter-domain dynamic trust management based on the pervasive environment. The approach makes a strict punishment to malicious actions and has good computing convergence and scalability. But the shortage of the PTM is that it obtains indirect trust value on average without taking the fuzziness, subjectivity and uncertainty into account.

Hsieh *et al.* [19] use cluster-based structure to ensure the security of wireless sensor networks which includes two modules: (1) the dynamic key authorization is adopted to prevent external malicious nodes from entering when a new cluster is established or a new node joins in the cluster. (2) The nodes in the cluster detect each other and different trust computing methods are formulated based on the different roles nodes act as. The approach is difficult to implement and exists weak computing convergence.

Marmol *et al*. [20] carry out a wide review of different trust models, provide some pre-standardization recommendations and propose an interface proposal for trust models. Lopez *et al*. [21] list the best practices that are essential for developing a good trust management system for WSN and make an analysis of the state of the art related to these practices. These two references make an excellent summary, propose many profound viewpoints and show an additional insight on the trust evaluation field. In addition, other protocols [22–24] address trust management methods in self-organization networks from different views.

## 3. Preliminaries

In this section, we describe the overall architecture of the NBBTE and various parameter definitions, including all sorts of trust factors, the fuzzy subset grade of membership functions, the calculation methods of trust worthiness based on the D-S theory.

### 3.1. Definitions of Trust Factors

The trust relationship, developing from sociology and economics, has strong subjectivity and uncertainty. The accurate and applicable trust metric is the precondition of establishing nodes’ trust degree. Some trust factors are analyzed from different viewpoints [15,16,20]. Inspired by these works, we define the composing elements of trust value according to the sensor nodes’ behavioral features to resolve common attacks and protect the network [1,21]. When node *i* evaluates the trust degree on the node *j*:

1. The factor of received packets rate *RPF*_i,j_(*t*): the change of node *j*’s received packets rate in period *t*.
2. The factor of successfully sending packets rate *SPF*_i,j_(*t*): node *j*’s successful rate of sending packets in period *t*.
3. The factor of packets forwarding rate *TPF*_i,j_(*t*): the relation between sent packets and received packets of node *j* in period *t.*
4. The factor of data consistency *CPF*_i,j_(*t*): the degree of difference for sending data packets between node *j* and its neighbor nodes.
5. The factor of time frequency *TFF*(*t*): time relativity of context content in period *t*.
6. The factor of node availability *HPF*_i,j_(*t*): the relativity between hello packets sent and ACK feedbacks received by node *i.*
7. Security grade SG: we define different security requirement according to the application fields of network (Battle field, emergency response, civil environment *etc*.).

### 3.2. Fuzzy Classification of Trust Relationship between Nodes

The trust degrees among sensor nodes are subjective and uncertain, which is decided by two aspects: (1) the classification of trust is not based on the ‘two-valued logic’ but ‘multiple-valued logic’. (2) A certain trust value may belong to various trust grades rather than merely one grade. Thus, fuzzy theory is more suitable to describe and deal with fuzzy notions compared with traditional mathematical models. In this paper, we provide an efficient approach to make a quantitative research on subjective trust relationship by making the use of membership degree and language variables in the fuzzy theory.

**Figure 1.**
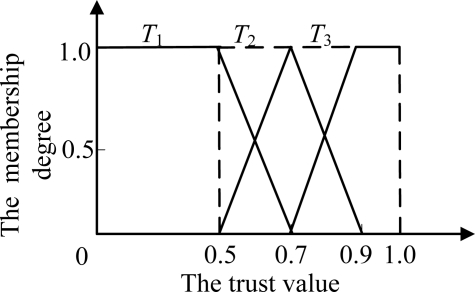
The membership function of node’s trust.

The specific fuzzy classification of nodes’ trust is as following. Firstly, the trust is divided into three grades: completely distrust, uncertain and completely trust state. Secondly, according to the three grades, it marks up three fuzzy subsets *T*_1_, *T*_2_ and *T*_3_ on the universe of nodes’ trust value T ([0,1]), as shown in Figure 1. The corresponding membership functions are *u*_1_(*t*), *u*_2_(*t*) and *u*_3_(*t*), *u*_1_(*t*) + *u*_2_(*t*) + *u*_3_(*t*) = 1.

### 3.3. Related Definitions and Rules in D-S Evidence Theory

The integrated trust value of one node given by its neighbor nodes cannot be simply established by weighted average due to the subjectivity of trust evaluation. While evidence theory proposed by Dempster and Shafer can briefly express the important conceptions, such as ‘uncertainty’ or ‘not-knowing’. In addition, it makes right judgments by efficiently integrating many-sided uncertain information. Motivated by this, we take advantage of D-S evidence theory to compute integrated trust evaluation of nodes.

D-S evidence theory [25] is based on the *Ω* set comprised by basic propositions which are both exclusive and exhaustive, termed identification frame. 2*^Ω^* is the power set of *Ω*, that is, the set of all the possible propositions based on *Ω*. Here we define *Ω* as {***T***, **−*T***}, where ***T*** and **−*T*** represent two trust states, namely credible and incredible. 2*^Ω^* is {**Φ**,{***T***},{**−*T***},{***T***, **−*T***}}, in which **Φ**, {***T***}, {**−*T***} and {***T***, **−*T***} represent respectively the empty set, the propositions of nodes’ ‘Trust’, ‘Distrust’ and ‘Uncertain’. There are definitions of basic reliability function *m* on 2*^Ω^*:2*^Ω^* → [0,1], reliability function *Bel*: 2*^Ω^* → [0,1] and likelihood function *Pl*: 2*^Ω^* → [0,1], satisfying the following equations:

(1) $$\{\begin{array}{l} m(\emptyset )=0\\ \sum_{A\subseteq \Omega }m(A)=1,A\neq \emptyset \end{array}$$

(2) $$\mathrm{Bel}(A)=\sum_{B\subseteq A}m(B),\forall A\subseteq \Omega$$

(3) $$\mathrm{Pl}(A)=1-\mathrm{Bel}(\bar{A}),\forall A\subseteq \Omega$$

where: *A* is named focal element, *m*(*A*) is the basic confidence level of *A*, representing how much the evidence support *A* to happen. *Pl(A*) and *Bel(A*) are the confidence level and likelihood of *A*, *Bel(****T***) = *m*({***T***}), *Pl*(***T***) = *m*({***T***}) + *m*({***T***, **−*T***}).

In our proposed algorithm, the nodes evaluate each other to acquire some of the seven trust degrees which are the probability forms. To reflect the subjectivity, uncertainty and fuzziness of trust, we transform probability forms of the trust degrees to the vector forms by using fuzzy membership function and D-S evidence theory, and each element in this vector represents the calculated basic confidence level to the ‘Distrust’, ‘Uncertain’, ‘Trust’ propositions of nodes.

As for the integrated trust value which should combine the direct and indirect trust values, we make a combination of multiple evidences according to the revised Dempster rule, which will be described in detail in Section 4.2.2.

## 4. NBBTE Algorithm

As described in the introduction, the NBBTE algorithm firstly establishes various trust factors depending on the interactions between neighbor nodes, which are observed by each other. Then the trust value is obtained by combining network security degree and correlation of time context. Secondly, it applies the fuzzy set theory to measure how much the trust value of node belongs to each trust degree. Finally, the integrated trust value of evaluation considering the recommendation of several neighbor nodes is acquired in accordance with the trust difference between evidences and the revised Dempster rule of combination.

### 4.1. Trust Evaluation Approach between Neighbor Nodes

**Figure 2.**
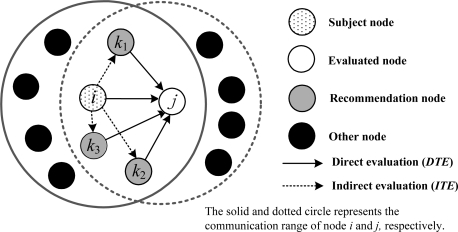
The recommendation trust relationship among nodes.

Trust depends on a subject’s (evaluating node) observation on the object (evaluated node) and third party recommendations. The WSNs’ features need a trust evaluation mechanism without central nodes, where neighbor nodes monitor each other. The subject obtains the trust value of objects according to both direct and indirect trust values. As shown in Figure 2, the node *i* is subject, which not only makes direct assessment of object *j*, but also makes indirect evaluation of object *j* through nodes *k*_1_, *k*_2_, *k*_3_.

#### 4.1.1. Trust Factors

It is essential to make quantitative and qualitative analysis of various factors which affect trust value in order to evaluate a node’s trust worthiness. In the following, we explain the establishment scheme of trust factors defined in Section 3.1. We assume that node *i* makes trust evaluation for node *j* and ACK mechanism is adopted. In other words, once the node receives a packet, it sends ACK feedback information to the sender.

(1) The factor of received packets rate *RPF*_i,j_(*t*): According to the assumption, if node *i* monitors node *j* to confirm how many common ACK packets node *j* sends, the ratio of packets received by node *j* can be obtained. According to the change of the ratio, we can know whether node *j* has response forging behavior. If the change maintains in the interval (−*ξ*, *ξ*) in different periods, node *j* works normally. The calculation equation is as follows (*RP*_ij_(*t*) represents the number of received packets):

(4) $${\mathrm{RPF}}_{i,j}\;(t)=\frac{{\mathrm{RP}}_{\mathrm{ij}}\;(t)-{\mathrm{RP}}_{\mathrm{ij}}\;(t-1)}{{\mathrm{RP}}_{\mathrm{ij}}\;(t)+{\mathrm{RP}}_{\mathrm{ij}}\;(t-1)}$$

(2) The factor of successfully sending packets rate *SPF*_i,j_(*t*): Assume that *j* sends packets to *k* who is beyond the communication scope of *i*. Although *i* cannot monitor the successfully sent packets rate of *j* directly in this situation, node *i* can monitor the number of the same packets sent by *j*. It’s known that every packet sent by nodes contains a time stamp and can be distinguished efficiently even if the packets have the same content. Thus, we can obtain the sending number of a certain packet according to different time stamps. The equation as follows (*SP*_ij_(*t*) is the needing number of sent packets, *SF*_ij_(*t*) is the repeating number of sent packets):

(5) $${\mathrm{SPF}}_{i,j}\;(t)=\frac{{\mathrm{SP}}_{\mathrm{ij}}\;(t)}{{\mathrm{SP}}_{\mathrm{ij}}\;(t)+{\mathrm{SF}}_{\mathrm{ij}}\;(t)}$$

(3) The rate of data forwarding *TPF*_i,j_(*t*): Multi-hop is usually necessary since most of nodes are impossible to communicate with the base station directly. If node *k* is beyond the communication range of node *i* and sends data packets to node *j*, node *i* cannot monitor the received packets number of node *j* directly and has to collect the ACK feedback information of node *j* to obtain the number of received packets. In order to distinguish the forwarding packets and the remained packets, an ACK packet which contains a special bit is constructed. Once node *j* receives a forwarding packet, it broadcasts an ACK packet above. Then node *i* can collect these ACK packets of node *j* to obtain the number of forwarding packets. According to the change rate of *TPF*_i,j_(*t*), it can efficiently avoid Sinkhole attack and Sybil attack, as well as identify whether the node is selfish. The equation as follows (*FP*_ij_(*t*) is the number of transmission packets):

(6) $${\mathrm{TPF}}_{i,j}\;(t)=\frac{{\mathrm{FP}}_{\mathrm{ij}}\;(t)-{\mathrm{FP}}_{\mathrm{ij}}\;(t-1)}{{\mathrm{FP}}_{\mathrm{ij}}\;(t)+{\mathrm{FP}}_{\mathrm{ij}}\;(t-1)}$$

(4) The consistency factor *CPF*_i,j_(*t*): The data packets have spatial correlation, that is, the packets sent among neighbor nodes are similar in the same area according to the application. So the consistency factor is introduced to prevent malicious nodes from modifying primary data packets. Node *i* acquires a packet transmitted by *j* randomly and makes the comparison with its own data. If the source node of this packet is in the same area of node *i* and the diversity rate maintains in the interval (−*ξ*, *ξ*), the number of accordant packets increases. Elsewise, if the source node does not belong to the area of node *i*, the consistency factor between node *i* and node *j* would not be adopted. The *CPF*_i,j_(*t*) equation as follows (*EP*_ij_(*t*) is the number of accordant packets, *NEP*_ij_(*t*) is the discordant one):

(7) $${\mathrm{CPF}}_{i,j}\;(t)=\frac{{\mathrm{EP}}_{\mathrm{ij}}\;(t)}{{\mathrm{EP}}_{\mathrm{ij}}\;(t)+{\mathrm{NEP}}_{\mathrm{ij}}\;(t)}$$

(5) Time factor *TFF*(*t*): Trust value has context relationship in time and content, and changes on the previous base. The size of time grade is dependent on the specific situation. If it is established too large, integrated trust value will be affected by history too heavily, which may cause errors in node evaluation. On the contrary, if it is established too small, trust value relies on a single period overly. Above all, we have the rules based on the security degree of networks. When the security degree is relatively high, *TFF*(*t*) = 0.8, relatively low, *TFF*(*t*) = 0.2, normally, *TFF*(*t*) = 0.5.

(6) The factor of availability *HPF*_i,j_(*t*): In some cases, the neighbor nodes are inaccessible due to wireless channel interference or bad environment. Concretely, node *i* sends HELLO packets for the detection whether they can be received by node *j*. If node *i* receive the ACK-HELLO packets from node *j*, it is proved that node *j* is accessible. The *HPF*_i,j_(*t*) equation as follows (*ACK*_ij_(*t*) is the amount of packets which have been responded, *NACK*_ij_(*t*) is the amount of packets which haven’t been responded):

(8) $${\mathrm{HPF}}_{i,j}\;(t)=\frac{{\mathrm{ACK}}_{\mathrm{ij}}\;(t)}{{\mathrm{ACK}}_{\mathrm{ij}}\;(t)+{\mathrm{NACK}}_{\mathrm{ij}}\;(t)}$$

(7) Security grade SG: According to specific application environment and scenario, the WSNs require different security degree based on different needs. For instance, there is a large difference between batter filed application and environmental monitor. When security requirement is high, SG = 3, relatively low, SG = 1; normally, SG = 2.

#### 4.1.2. Trust Calculation between Neighbor Nodes

As shown in Figure 2, trust evaluation of the subject to object includes direct evaluation *DTE* and indirect evaluation *ITE*. For example, if node *j* is evaluated, node *i* not only obtains the trust value of node *j* by itself directly but also through *k*_1_, *k*_2_ and *k*_3_ indirectly. And then node *i* integrates the two kinds of trust values to get an integrated one (for node *j*). Trust in the network has the feature ‘hard to acquire and easy to lose’. There are two trust initialization strategies: the pessimistic and the optimistic strategy. The pessimistic strategy can eliminate the possibility that the malicious node creates a new identity and impersonates a new node to rejoin in the network with the purpose of throwing away its bad trust value [21]. While the optimistic one has the foundation of completely trust at the beginning of network deployment, and is propitious to the quick network expansion. Therefore, in the initial period, trust value between neighbor nodes is depended on the factual application. Then, the trust of nodes changes gradually depending on behavior, history and time.

##### (1) *DTE* Evaluation Approach

For ensuring the reasonability of trust evaluation approach, different action parameters *μ* must be constituted in the different periods. For instance, in one period, node *i* and *j* interact with each other 10,000 times and the satisfactory actions are 5,000. In another period, they interact 1,000 times and the satisfactory actions are 500. Both periods have a satisfactory action rate of 0.5, obviously, the former is more believable. Therefore, we have the action parameter equation as follows:

(9) $$\mu =\frac{S_{\mathrm{ij}}\;(t)/\left(F_{\mathrm{ij}}\;(t)+S_{\mathrm{ij}}\;(t)\right)}{S_{\mathrm{ij}}\;(t-1)/\left[F_{\mathrm{ij}}\;(t-1)+S_{\mathrm{ij}}\;(t-1)\right]}$$

where: *S_ij_*(*t*) is the number of success, *F_ij_*(*t*) is the number of failure. In accordance with the trust factors in Section 4.1.1, combining with node recourse and network function environment, the direct trust value *DTE*_i,j_(*t*) is:

(10) $$\begin{array}{c} {\mathrm{DTE}}_{i,j}\;(t)=\mu^{\mathrm{SG}}\times \mathrm{TFF}(t)\times [w_{1}\times \left(1-\left|{\mathrm{RPF}}_{i,j}\;(t)\right|\right)+w_{2}\times \left|{\mathrm{SPF}}_{i,j}\;(t)\right|+w_{3}\times \left(1-\left|{\mathrm{TPF}}_{i,j}\;(t)\right|\right)\\ +w_{4}\times \left|{\mathrm{CPF}}_{i,j}\;(t)\right|+w_{5}\times \left|{\mathrm{HPF}}_{i,j}\;(t)\right|]+(1-\mathrm{TFF}(t))\times {\mathrm{DTE}}_{i,j}\;(t-1) \end{array}$$

where: *w*_1_, *w*_2_, *w*_3_, *w*_4_, *w*_5_ are trust factor weights. They are independent with each other and adjustable with different practical applications and satisfy:

(11) $$w_{1}+w_{2}+w_{3}+w_{4}+w_{5}=1$$

##### (2) *ITE* Evaluation Approach

Evaluation subject *i* collects other nodes' opinions on the evaluated object *j* as the indirect recommendation values. In order to decrease network communication payload and avoid trust recycle recursion, the recommendation values are confined to direct trust value of the common neighbors owned by both node *i* and *j*. As shown in the Figure 2, node *i* can only get the trust recommendation of *j* from *k*_1_, *k*_2_ and *k*_3_. Trust recommendation value is as follows according to the principle of trust transfer decline:

(12) $${\mathrm{ITE}}_{i,j}\;(t)={\mathrm{DTE}}_{i,k}\;(t)\times {\mathrm{DTE}}_{k,j}\;(t)$$

where *k* is the common neighbor of both node *i* and *j*.

### 4.2. The Method of Integrated Trust Value

Generally, the evaluated object has many neighbor nodes, and every neighbor has the direct trust value and indirect trust value of the evaluated object. To realize the fuzziness, subjectivity and uncertainty of trust evaluation, the D-S evidence theory method is adopted to obtain the integrated trust value instead of the simple weighted-average one.

The trust value of evaluation made by subject to object is obtained in Section 4.1. Based on it, we use the membership function of nodes’ trust classification to calculate the basic confidence level of trust evidence to the ‘Distrust’, ‘Uncertain’, ‘Trust’ propositions of nodes. Then the integrated trust value of the object is acquired by composing the direct and indirect trust value which is based on the revised Dempster integration rules.

#### 4.2.1. Trust Vector

In the process of integrating trust, the trust level of node *i* to *j* is indicated in vector form. The direct, indirect and integrated trust vectors of node *i* to *j* are:

(13) $$\begin{array}{l} {\mathrm{VDTE}}_{i,j}\;(t)=\left(m_{i,j}^{D}(\{-T\}),m_{i,j}^{D}(\{T,-T\}),m_{i,j}^{D}(\{T\})\right)\\ {\mathrm{VITE}}_{i,j}^{k_{1}}\;(t)=\left(m_{i,j}^{k_{1}}\;(\{-T\}),m_{i,j}^{k_{1}}\;(\{T,-T\}),m_{i,j}^{k_{1}}\;(\{T\})\right)\\ \ldots \\ {\mathrm{VITE}}_{i,j}^{k_{l}}\;(t)=\left(m_{i,j}^{k_{l}}\;(\{-T\}),m_{i,j}^{k_{l}}\;(\{T,-T\}),m_{i,j}^{k_{l}}\;(\{T\})\right)\\ {\mathrm{VAT}}_{i,j}\;(t)=\left(m_{i,j}\;(\{-T\}),m_{i,j}\;(\{T,-T\}),m_{i,j}\;(\{T\})\right) \end{array}$$

According to Equations (10) and (12), the probability forms of direct and indirect trust value of object *j* what the subject *i* gets are defined as *DTE*_i,j_(*t*) and *ITE*_i,j_(*t*). Using the corresponding membership functions in Section 3.2, we compute the fuzzy membership grades of node *j* to the ‘Distrust’, ‘Uncertain’ and ‘Trust’ grade. The fuzzy membership grades of *DTE*_i,j_(*t*), *u*_1_^D^, *u*_2_^D^, *u*_3_^D^, and the grades of *ITE*_i,j_(*t*), *u*_1_^I^, *u*_2_^I^, *u*_3_^I^ are calculated by:

(14) $$\begin{array}{lll} \{\begin{array}{l} u_{1}^{D}=u_{1}\;({\mathrm{DTE}}_{i,j}\;(t))\\ u_{2}^{D}=u_{2}\;({\mathrm{DTE}}_{i,j}\;(t))\\ u_{3}^{D}=u_{3}\;({\mathrm{DTE}}_{i,j}\;(t)) \end{array} & \text{and} & \{\begin{array}{l} u_{1}^{I}=u_{1}\;({\mathrm{ITE}}_{i,j}\;(t))\\ u_{2}^{I}=u_{2}\;({\mathrm{ITE}}_{i,j}\;(t))\\ u_{3}^{I}=u_{3}\;({\mathrm{ITE}}_{i,j}\;(t)) \end{array} \end{array}$$

If we regard the membership function of nodes' trust classification as the basic confidence function of propositions {**−*T***}{***T***, **−*T***}{***T***}, *u*_1_(*t*), *u*_2_(*t*) and *u*_3_(*t*) respectively stand for the support levels of trust evidences to the ‘Distrust’, ‘Uncertain’ and ‘Trust’ grade. At this point, *m*_i,j_^D^({**−*T***}), *m*_i,j_^D^({***T***, **−*T***}) and *m*_i,j_^D^({***T***}), the components of direct trust vector *VDTE*_i,j_(*t*), are respectively equal to *u*_1_^D^, *u*_2_^D^ and *u*_3_^D^. Similarly, *m*_i,j_^k^({**−*T***}), *m*_i,j_^k^({***T***, **−*T***}) and *m*_i,j_^k^ ({***T***}), the components of indirect trust vector *VITE*_i,j_(*t*), correspond to *u*_1_^I^, *u*_2_^I^ and *u*_3_^I^, respectively.

#### 4.2.2. Integrating the Trust Value

In this section, we combine the direct and indirect trust values of node *j* to get the integrated trust value. The integration process is in accordance with the revised Dempster combination rule, which is described in detail in the following.

Assume that *Bel*_1_ and *Bel*_2_ are two trust degree functions that on the same recognizing frame *Ω*, their basic trust degree function are *m*_1_ and *m*_2_. If ∑_X∩Y=∅︀_ *m*_1_(*X*) × *m*_2_(*Y*) < 1, the orthogonal of *Bel*_1_ and *Bel*_2_ is *Bel* = *Bel*_1_ θ *Bel*_2_, where *A* ∈ Ω. And *m*, the basic trust degree function of *Bel* can be expressed as follows:

(15) $$\{\begin{array}{l} m(A)=m_{1}\;(A)⊕m_{2}\;(A)=\frac{\sum_{X\cap Y=A}m_{1}\;(X)\times m_{2}\;(Y)}{\begin{array}{l} 1-K\\ A\neq \emptyset ,A\subseteq \Omega \end{array}}\\ m(\emptyset )=0 \end{array}$$

(16) $$K=\sum_{X\cap Y=\emptyset }m_{1}\;(X)\times m_{2}\;(Y)$$

As shown in Figure 2, at the end of period *t*, node *i* makes an integrated trust evaluation on node *j*. From human’s action model and the trust’s subjectivity, we know that the weight of direct and indirect trust values shouldn’t be the same. The weight of *VDTE*_j_(*t*) and *VITE*_j_(*t*) should be modified. Assume the weight of direct trust value is 1, and the indirect evidence’s weight is changed according to the similarity between direct and indirect trust value. On this basis, we use the Equations (15) and (16) to calculate the integrated trust value *VAT*_i,j_(*t*) (subject *i* to object *j*).

Next, the course of modification evidential weight is described. If node *i* gets *l* indirect trust values to evaluated node *j*, the difference between any indirect and direct evidence are as follows:

(17) $$D_{k_{n},\;i}\;(t)=\sqrt{\frac{1}{2}{\left(\vec{m_{i,j}^{k_{n}}}-\vec{m_{i,j}^{D}}\right)}^{T}D\left(\vec{m_{i,j}^{k_{n}}}-\vec{m_{i,j}^{D}}\right)}=\sqrt{\frac{1}{2}({\left\|m_{i,j}^{k_{n}}\right\|}^{2}+{\left\|m_{i,j}^{D}\right\|}^{2}-2\langle \vec{m_{i,j}^{k_{n}}},\vec{m_{i,j}^{D}}\rangle )}$$

The $\vec{m_{i,j}^{k_{n}}}$ and $\vec{m_{i,j}^{D}}$, defined in the Equation (13), are indirect and direct trust vector, respectively. When the evidence difference *D_k_n_,i_* > *ξ*, it usually means that a malicious node is sending bogus information. Then, the similarity parameter between different evidences is modulated according to Equations (13) and (17):

(18) $$S_{k_{n},i}\;(t)=1-D_{k_{n},i}\;(t)$$

From the Equation (18), the modified weight of indirect evidence is:

(19) $$\left\{ \begin{array}{l} \Delta m_{i,j}^{k_{n}}\;(\{T\})=S_{k_{n},\;i}\;(t)*m_{i,j}^{k_{n}}\;(\{T\})\\ \Delta m_{i,j}^{k_{n}}\;(\{-T\})=S_{k_{n},\;i}\;(t)*m_{i,j}^{k_{n}}\;(\{-T\})\\ \Delta m_{i,j}^{k_{n}}\;(\{T,-T\})=1-m_{i,j}^{k_{n}}\;(\{T\})-m_{i,j}^{k_{n}}\;(\{-T\}) \end{array}\right.$$

Above all, the indirect evidence can be modified as:

(20) $$\Delta \mathrm{VIT}E_{i,j}^{k_{n}}\;(t)=\left(\Delta m_{i,j}^{k_{n}}\;(\{-T\}),\Delta m_{i,j}^{k_{n}}\;(\{T,-T\}),\Delta m_{i,j}^{k_{n}}\;(\{T\})\right)$$

According to Equations (10), (15), (16) and (20), the integrated trust value is as follows:

(21) $$m_{i,j}\;(A)=m_{i,j}^{D}(A)⊕\frac{1}{l}\sum_{n=1}^{l}\Delta m_{i,j}^{k_{n}}\;(A)$$

Finally [26], the eventual basic confidence assignment value is established by Equation (21), if the decision model satisfies:

(22) $$\left\{ \begin{array}{l} m_{i,j}\;(\{T\})-m_{i,j}\;(\{-T\})>ɛ\\ m_{i,j}\;(\{T,-T\})<\theta \\ m_{i,j}\;(\{T\})>m_{i,j}\;(\{T,-T\}) \end{array}\right.$$

Then main subject node *i* regards node *j* as trust, and add node *j* into its trustworthiness list. In like manner, node *j* can be marked ‘Uncertain’ or ‘Distrust’.

## 5. Simulations

**Figure 3.**
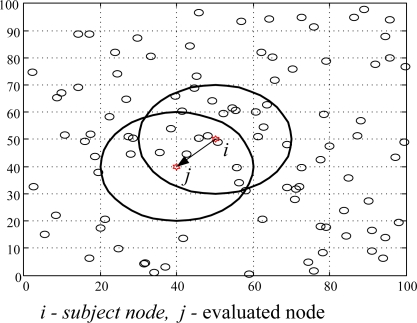
The distribution map of random nodes.

We used MatLab as simulation tool to analyze the performances of the NBBTE algorithm in this section, which includes: trust evaluation of credible nodes, incredible nodes, and malicious nodes, as well as the influence of factors on the trust evaluation. The concrete simulation scene is a square area of 100 m × 100 m, with 100 randomly deployed nodes. Here the communication radius is 20 m. We assume node *i* is subject node and node *j* is evaluated node, overlapping part of two circles is the common neighbor of evaluation and evaluated node (Figure 3). From the viewpoint of rigorous security requirement, the pessimistic initialization strategy of trust value is adopted, and the initial trust state of nodes is set as incredible. Some parameters vary with the scenes and the purposes of experiment and will be explained in detail.

### 5.1. Analysis of Trust Evaluation

Preferences: In this section, *w*_1_ = *w*_2_ = *w*_3_ = *w*_4_ = *w*_5_ = 0.2, security degree SG = 1, behavioral coefficient *μ* = 1, time factor *TFF*(*t*) changes with nodes’ trust value. When the trust value of node *j* increases gradually, *TFF*(*t*) = 0.2, in contrast, *TFF*(*t*) = 0.8. If all the change rates of trust factors are in the range of 10% ∼ 20%, node *j* is thought to be a non-malicious node, else if the change rates are 50%, node *j* is abnormal and has malicious or self actions.

**Figure 4.**
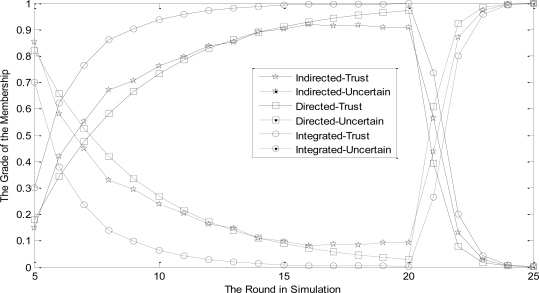
The membership grade of the distrustful actions.

The change tendency of node membership grade to three basic trust degree functions reflects the effectiveness of revised D-S evidence theory. In this section, the character of ‘trust is hard to acquire and easy to lose’ should be expressed. Simulation results are shown in Figure 4.

Firstly, we assume the actions of each node in network are normal. In Figure 4, when the trust evaluation system tends to be stable, trust value of node *j* increases slowly. Therefore, its membership grade to ‘Trust’ increases gradually and the one to ‘uncertain’ accordingly decreases. Meanwhile, integrated trust value is higher than both direct and indirect trust value. That is because the proposed scheme adopts D-S theory to integrate trust values of nodes which lends the reality of node *j* to be represented better. On the contrary, using the weighted-average method, integrated trust value is lower than direct trust value, which fails to show the advantage of trust value integration.

Secondly, in order to reflect that the NBBTE can achieve the character of ‘trust is hard to acquire and easy to lose’, we assume the actions of each node in network are abnormal when the trust value measure up to the peak. It can be seen from Figure 4 that the evaluation model reflects nodes’ change in acutely behavior and has more sensitive change to malicious actions. The trust value increases slowly for fifteen periods when a node collaborates well with others. However, when the node begins to uncooperative, its trust value decreases quickly to nearly zero in only four periods. This result verifies the NBBTE represents the character ‘trust is hard to acquire and easy to lose’.

### 5.2. The Influence of Malicious Nodes

Preferences: the number of malicious nodes in the common neighbors of both node *i* and *j* are 10%, 20% and 30%, respectively. Other preferences are the same as Section 5.1. Most existing trust evaluation algorithms adopt the average methods to calculate the trust value of evaluated nodes, which can not reflect the actual contribution of different subject nodes (direct and indirect nodes).

**Figure 5.**
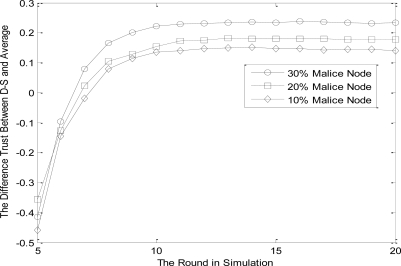
The comparison between NBBTE and weighed-average method.

In this section, the function of NBBTE algorithm is assessed by introducing different proportions of malicious nodes, which send bogus indirect trust values. The trust difference between average method and the proposed scheme is used to show the advantages of NBBTE algorithm. Assume that evaluation subject *i* is credible and the membership degrees of malicious nodes are opposite compared with normal ones. We can see from Figure 5 that the two schemes in membership degree to ‘TRUST’ tend to be stable gradually with evaluation time, but the more the malicious nodes, the greater the difference. That’s because that NBBTE algorithm applies the revised Dempster rule of combination, which modulates indirect weight according to the evidence differences between direct and indirect trust value. Thus, the proposed scheme takes fuzziness and subjectivity of trust in consideration. With the increasing of malicious nodes, weight modulation shows more advantage.

## 6. Conclusions

In order to identify selfish and malicious nodes efficiently and solve the security problems for node failure or capture in WSNs, this paper proposes a trust evaluation algorithm based on behavior strategy banding D-S belief theory, which successfully underlines the fuzziness, subjectivity and usability of trust. The core works of the algorithm (NBBTE) include the following parts:

Establish trust factors by considering network’s practical application environment. Then make both quantitative and qualitative analysis to calculate the direct and indirect trust value.

Obtain the membership degree of trust value to trust grade by fuzziness set theory and accordingly form the basic input vector of evidence theory.

Revise the Dempster rule of combination and modulate similarity coefficient in accordance with differences between indirect and direct evidences to integrate the trust value of evaluated node.

At the end, the simulations show that the scheme can assess nodes’ trustworthiness efficiently accounting for subjectivity, uncertainty and fuzziness of trust evaluation. Furthermore, it can represent the feature that ‘trust is hard to acquire and easy to lose’ and improve the security of networks.

The process of trust evaluation may need excess energy and time costs due to the cooperation and communication with neighbors, and the memory costs also increase with the parameter numbers, algorithm precision and network density. However, to enhance the network security, a compromise has to be made between the trust and the resource consumption. And we believe that the resource constraint of nodes is likely to be resolved in the future due to the technical development.

As future work a real experiment is being designed to estimate the performance of algorithm. Moreover, applications based on node trust are being considered, such as routing, data aggregation and so on.
